# Rate, characteristics, and factors associated with high emergency department utilization

**DOI:** 10.1186/1865-1380-7-9

**Published:** 2014-02-05

**Authors:** M Christien van der Linden, Crispijn L van den Brand, Naomi van der Linden, Anna HJH Rambach, Caro Brumsen

**Affiliations:** 1 Emergency Department, Medical Centre Haaglanden, P.O. Box 432, 2501, CK The Hague, The Netherlands; 2 Institute for Medical Technology Assessment, Erasmus University Rotterdam, P.O. Box 1738, 3000, DR Rotterdam, The Netherlands; 3 Department of Internal Diseases, Medical Centre Haaglanden, P.O. Box 432, 2501, CK The Hague, The Netherlands

**Keywords:** Emergency department, Emergency service, Frequent visits, Hospital

## Abstract

**Background:**

Patients with high emergency department (ED) utilization account for a disproportionate number of ED visits. The existing research on high ED utilization has raised doubts about the homogeneity of the frequent ED user. Attention to differences among the subgroups of frequent visitors (FV) and highly frequent visitors (HFV) is necessary in order to plan more effective interventions.

In the Netherlands, the incidence of high ED utilization is unknown. The purpose of this study was to investigate if the well-documented international high ED utilization also exists in the Netherlands and if so, to characterize these patients. Therefore, we assessed the proportion of FV and HFV; compared age, sex, and visit outcomes between patients with high ED utilization and patients with single ED visits; and explored the factors associated with high ED utilization.

**Methods:**

A 1-year retrospective descriptive correlational study was performed in two Dutch EDs, using thresholds of 7 to 17 visits for frequent ED use, and greater than or equal to 18 visits for highly frequent ED use.

**Results:**

FV and HFV (together accounting for 0.5% of total ED patients) attended the ED 2,338 times (3.3% of the total number of ED visits). FV and HFV were equally likely to be male or female, were less likely to be self-referred, and they suffered from urgent complaints more often compared to patients with single visits. FV were significantly older than patients with single visits and more often admitted than patients with single visits. Several chief complaints were indicative for frequent and highly frequent ED use, such as shortness of breath and a psychiatric disorder.

**Conclusions:**

Based on this study, high ED utilization in the Netherlands seems to be less a problem than outlined in international literature. No major differences were found between FV and HFV, they presented with the same, often serious, problems. Our study supports the notion that most patients with high ED utilization visit the ED for significant medical problems.

## Background

Patients with high emergency department (ED) utilization, also called “frequent visitors” (FV) account for a disproportionate number of ED visits [1,2]. Patients with high ED utilization are a well-studied group in the literature [1-17]. The definition of high ED utilization is debatable, ranging from 2 to more than 12 visits per year [2]. Contrary to popular belief, FV are more likely to be admitted and more likely to die in the ED, suggesting that FV are generally sicker than infrequent visitors [1,2,7]. FV represent severe psychosocial and medical vulnerability [5] and they are often heavy users of other health and social services [18].

The existing research on high ED utilization has raised doubts about the homogeneity of the FV [15,19,20]. Although FV are known to be a vulnerable population with a poor health status [14], the opposite may be true for the highest frequency visitors. Highly frequent visitors (HFV), defined as patients with 20 or more visits per year, were found to be less ill or injured than patients with single visits [15]. Attention to differences among the subgroups of FV and HFV is necessary in order to plan more effective interventions [20].

### Importance

In the Netherlands, the incidence of high ED utilization is unknown. The purpose of this study was to investigate whether the well-documented international high ED utilization also exists in the Netherlands and if so, to characterize these patients.

Therefore, we assessed the proportion of FV and HFV; compared age, sex, and visit outcomes between patients with high ED utilization and patients with single ED visits; and explored the factors associated with high ED utilization.

## Methods

A retrospective, descriptive, correlational study was performed in two ED locations in the Netherlands: a level one ED in an inner-city trauma centre and a level three ED in a hospital located in a small city. High ED utilization was defined according to thresholds developed by Doupe et al. [6]: patients with 7 to 17 visits in 2012 were considered to be FV and those with 18 or more visits in 2012 were considered to be HFV.

Variables collected from the hospitals’ database were based on previous research regarding high ED utilization and included age [2,12,21], sex [21], arrival time, arrival transport mode (ambulance or not) [21], referral source (self-referred or non-self-referred), chief complaint [3,10,13,16], triage level [2,17], and visit outcome (admission, leaving without being seen by a doctor (LWBS), or death at the ED) [3,10,21]. Arrival time was categorized in three shifts: day shift (7.30 to 15.29 h), evening shift (15.30 to 22.59 h), and night shift (23.00 to 7.29 h). Chief complaints were identified from the triage notes for each visit. Chief complaints occurring at least 500 times per year were categorized as such, while chief complaints occurring less than 500 times were categorized as ‘Other’. Triage levels were assigned according to the 5-level Manchester Triage System, where: 1. immediate, 2. very urgent, 3. urgent, 4. standard, and 5. non-urgent [22]. In the analysis, triage levels were combined because of small numbers of patients with levels 1 and 5, to immediate/very urgent, urgent, and standard/non-urgent. Institutional review board exemption was granted.

### Analysis

The proportions of FV and HFV were summarized using descriptive statistics. Age, sex, visit outcomes, and triage level were compared between the patients with high ED utilization (FV and HFV) and patients with a single ED visit, using *t*-tests (age) and χ^2^ tests (sex, hospital admission, LWBS, death, triage level). The associations of visit characteristics with the presence of frequent ED use and highly frequent ED use were analysed using logistic regression. The variables included in the models were arrival time (reference: day shift), arrival with ambulance, self-referral, chief complaint (each medical complaint versus rest of the patients), and triage level (reference: immediate/very urgent).

Two separate logistic regression models were developed, comparing frequent ED use with single ED use, and highly frequent ED use with single ED use. In both models, the ED visit was used as a unit of analysis. Adjusted odds ratios (ORs) are provided with their 95% confidence intervals (CI) to indicate the likelihood of frequent use or highly frequent use for each explanatory variable adjusted for the other variables. The calibration and overall discriminative capacity of the final models were assessed with the Hosmer-Lemeshow test and the area under the receiver operating curve (AUC ROC) analysis, respectively [23]. Data were analysed using Predictive Analytics Soft Ware, version 18 (Chicago, IL, USA).

## Results

During the 1-year study period, 71,565 consultations were registered at the two EDs, of which 50,155 were at the level one ED and 21,410 at the level three ED. These visits were paid by 51,272 different individuals. Of these 51,272 patients, 38,959 patients visited the ED a single time during the study period.

### The proportion of frequent visitors (FV) and highly frequent visitors (HFV)

During the study year, 244 patients attended the ED 7 times or more (Table 1): 95% of them (n = 232) attended 7 to 17 times on 2,075 occasions and were considered FV.

**Table 1:** Number of ED visits (244 patients representing 2,338 ED visits)

**No. of ED visits**	**n (total 244)**	**%**	**Cumulative %**
7	90	36.9	36.9
8	39	16.0	
9	36	14.8	
10	22	9.0	76.6
11	12	4.9	
12	12	4.9	
13–17	21	8.6	95.1
18	2	0.8	
20	4	1.6	
22	2	0.8	
23	3	1.2	99.6
34	1	0.4	100

The remaining 12 patients attended the ED 18 times or more during the study year and were categorized as HFV (attending the ED on 263 occasions). FV and HFV (together accounting for 0.5% of total ED patients) attended the ED 2,338 times (3.3% of the total number of ED consultations).

### Patient characteristics and visit outcomes

Results are presented for both EDs together, since no statistically significant differences in the results were found between the two EDs. Patient characteristics are shown in Table 2. FV were equally likely to be male or female and were significantly older than patients with single visits (mean age 47.5 vs. 39.0 years, *P* <0.001). Among FV, most patients (n = 75, 32.3%) were in the age category of 45 to 64 years (data not shown). No differences were found in sex and mean age and between the HFV and the patients with single visits.

**Table 2:** Patients with one ED visit (n = 38,959) compared with patients with high ED utilization (n = 244)

	**Patients with one ED visit (n = 38,959)**	**Patients with high ED utilization (n = 244)**	***P *value**
**Frequent visitors (n = 232)**			
Sex, male (n, %)	19,788 (50.8)	128 (55.2)	0.183
Age (mean, standard deviation)	39.0 (23.1)	47.5 (20.5)	<0.001
**Highly frequent visitors (n = 12)**			
Sex, male (n, %)	19,788 (50.8)	7 (58.3)	0.601
Age (mean, standard deviation)	39.0 (23.1)	48.3 (17.9)	0.161

Visit outcomes are shown in Table 3. Consultations of FV ended in admission significantly more often than consultations of patients with single visits (24.5% vs. 15.9%, *P* <0.001). LWBS occurred more often during visits of FV. No differences in visit outcomes were found between HFV and patients with single visits. There was no mortality in the patients with frequent or highly frequent ED use.

**Table 3:** Disposition of patients with high ED utilization (compared with single visit patients (n = 38,959)

	**Visits of patients with a single visit (n = 38,959)**	**Visits of FV (n = 2,075)**	***P *value**	**Visits of HFV (n = 263)**	***P *value**
			Single visits – visit of FV*		Single visits – visits of HFV*
**Admitted** [n (%)]	6,189 (15.9)	509 (24.5)	<0.001	49 (18.6)	0.225
**LWBS** [n (%)]	404 (1.0)	46 (2.2)	<0.001	1 (0.4)	0.294
**Died** [n (%)]	31 (0.1)	0	-	0	-

*χ^2^ tests.

### Factors associated with high ED utilization

Factors associated with high ED utilization are shown in Table 4 (attendances of FV) and Table 5 (attendances of HFV). Because no notable differences were found between the two EDs in factors associated with high ED utilization, results are presented for both EDs combined.

**Table 4:** Factors associated with frequent ED use

	**Frequent ED use (n = 2,075)**	**Single ED visits (n = 38,959)**	**OR (95% CI)**^**1, 2 **^**frequent ED use**	**OR (95% CI)**^**1, 2 **^final model, frequent ED use*
**Arrival time**				
Day shift	888 (42.8)	17,100 (43.9)	Reference	Reference
Evening shift	812 (39.1)	17,078 (43.8)	1.02 (0.92, 1.13)	1.00 (0.90, 1.10)
Night shift	375 (18.1)	4,781 (12.3)	1.42 (1.24, 1.62)	1.38 (1.21, 1.57)
**Arrival with ambulance**	339 (16.3)	4,736 (12.2)	0.91 (0.79, 1.05)	-
**Self-referred**	1,108 (53.4)	26,643 (68.4)	0.70 (0.63, 0.78)	0.70 (0.64, 0.77)
**Chief complaint** [n (%)]				
Limb problems	235 (11.3)	10,028 (25.7)	0.48 (0.39, 0.59)	0.46 (0.39, 0.54)
Wounds & local infections	147 (7.1)	4,977 (12.8)	0.60 (0.48, 0.76)	0.58 (0.48, 0.70)
Ear/Nose/Sore throat	14 (0.7)	895 (2.3)	0.33 (0.19, 0.57)	0.31 (0.18, 0.54)
Shortness of breath	176 (8.5)	1,376 (3.5)	2.32 (1.84, 2.92)	2.15 (1.79, 2.59)
Abdominal pain	402 (19.4)	3,208 (8.2)	2.36 (1.94, 2.87)	2.21 (1.92, 2.55)
Chest pain	220 (10.6)	2,784 (7.1)	1.46 (1.16, 1.83)	1.37 (1.16, 1.62)
Unwell patient	126 (6.1)	2.146 (5.5)	1.08 (0.84, 1.38)	-
Head ache & head injury	56 (2.7)	1,919 (4.9)	0.56 (0.41, 0.77)	0.52 (0.39, 0.69)
Psychiatric disorders	110 (5.3)	541 (1.4)	3.36 (2.55, 4.41)	3.03 (2.41, 3.83)
Back pain	36 (1.7)	705 (1.8)	1.04 (0.72, 1.52)	-
Severe trauma	9 (0.4)	753 (1.9)	0.21 (0.11, 0.42)	0.19 (0.10, 0.37)
Rashes	21 (1.0)	522 (1.3)	0.82 (0.51, 1.31)	-
Urinary tract problems	60 (2.9)	447 (1.1)	2.61 (1.90, 3.59)	2.46 (1.84, 3.27)
Worried parent	2 (0.1)	346 (0.9)	0.12 (0.03, 0.47)	0.11 (0.03, 0.45)
Eye problems	10 (0.5)	901 (2.3)	0.24 (0.13, 0.46)	0.23 (0.12, 0.43)
Pregnancy problems	21 (1.0)	412 (1.1)	1.02 (0.64, 1.63)	-
Other	201 (9.7)	2,901 (7.4)	1.32 (1.06, 1.64)	1.26 (1.05, 1.49)
**No triage**	229 (11.0)	4,098 (10.5)	-	-
**Triage level**				
Immediate & very urgent	403 (20.2)	5,286 (14.0)	Reference	-
Urgent	752 (37.7)	11,749 (31.1)	0.95 (0.83, 1.10)	-
Standard & non-urgent	840 (42.1)	20,768 (54.9)	1.01 (0.87, 1.19)	-
No triage	80 (3.9)	1,156 (3.0)	-	-

^1^Adjusted for other variables by logistic regression, OR >1 indicate an increased risk of frequent visit.

^2^Model based on 39,798 observations due to 1,236 missing values.

*Hosmer-Lemeshow test <0.001, AUC ROC 0.70 (0.69, 0.71).

**Table 5:** Factors associated with highly frequent ED use

	**Visit of HFV (n = 263)**	**Visits of patients with one ED visit (n = 38,959)**	**OR (95% CI)**^**1,2 **^**highly frequent ED use**	**OR (95% CI) )**^**1,2 **^final model, highly frequent ED use*
**Arrival time**				
Day shift	102 (38.8)	17,100 (43.9)	Reference	Reference
Evening shift	97 (36.9)	17,078 (43.8)	1.05 (0.79, 1.40)	1.05 (0.79, 1.40)
Night shift	64 (24.3)	4,781 (12.3)	1.73 (1.24, 2.42)	1.71 (1.23, 2.40)
**Arrival with ambulance**	78 (29.7)	4,736 (12.2)	2.02 (1.43, 2.85)	1.97 (1.40, 2.79)
**Self-referred**	119 (45.2)	26,643 (68.4)	0.68 (.050, 0.93)	0.67 (0.49, 0.91)
**Chief complaint** [n (%)]				
Limb problems	25 (9.5)	10,028 (25.7)	0.55 (0.29, 1.04)	-
Wounds & local infections	27 (10.3)	4,977 (12.8)	1.22 (0.65, 2.28)	-
Ear/Nose/Sore throat	1 (0.4)	895 (2.3)	0.25 (0.03, 1.92)	-
Shortness of breath	41 (15.6)	1,376 (3.5)	4.49 (2.48, 8.11)	9.17 (6.10, 13.78)
Abdominal pain	42 (16.0)	3,208 (8.2)	2.22 (1.24, 3.98)	4.47 (3.01, 6.65)
Chest pain	30 (11.4)	2,784 (7.1)	1.87 (0.99, 3.53)	3.88 (2.43, 6.19)
Unwell patient	8 (3.0)	2.146 (5.5)	0.46 (0.19, 1.09)	-
Head ache & head injury	2 (0.8)	1,919 (4.9)	0.16 (0.04, 0.70)	-
Psychiatric disorders	20 (7.6)	541 (1.4)	3.49 (1.75, 6.96)	7.23 (4.22, 12.40)
Back pain	0	705 (1.8)	-	-
Severe trauma	0	753 (1.9)	-	-
Rashes	1 (0.4)	522 (1.3)	0.41 (0.05, 3.09)	-
Urinary tract problems	36 (13.7)	447 (1.1)	14.68 (8.04, 26.81)	29.43 (19.25, 44.98)
Worried parent	0	346 (0.9)	-	-
Eye problems	1 (0.4)	901 (2.3)	0.27 (0.04, 2.06)	-
Pregnancy problems	0	412 (1.1)	-	-
Other	12 (4.6)	2,901 (7.4)	0.73 (0.35, 1.55)	-
**No triage**	17 (6.5)	4,098 (10.5)	-	-
**Triage level**				
Immediate & very urgent	53 (20.2)	5,286 (14.0)	Reference	Reference
Urgent	123 (46.9)	11,749 (31.1)	1.42 (0.99, 2.02)	1.48 (1.04, 2.11)
Standard & non-urgent	86 (32.8)	20,768 (54.9)	1.05 (0.68, 1.61)	1.16 (0.76, 1.76)
No triage	1 (0.4)	1,156 (3.0)	-	-

^1^Adjusted for other variables by logistic regression, OR >1 indicate an increased risk of frequent visit.

^2^Model based on 38,065 observations due to 1,157 missing values.

*Hosmer-Lemeshow test 0.14, AUC ROC 0.79 (95% CI, 0.76–0.82).

Most of the FV and HFV arrived during the day shift or evening shift. However, when corrected for other variables, arriving during the night shift was indicative for high ED utilization. Self-referral was less likely to occur among FV and HFV compared to the patients with single visits. Several chief complaints were indicative for frequent and highly frequent ED use, namely shortness of breath, abdominal pain, urinary tract problems, and psychiatric disorders. HFV arrived by ambulance more often than patients with single visits.

Both FV and HFV were assigned to the non-urgent or standard triage level significantly less often than patients with single visits (42.1% of the FV were non-urgent or standard and 32.8% of the HVF were non-urgent or standard, compared with 54.9% of the patients with single visits, *P* <0.001).

The Hosmer-Lemeshow goodness-of-fit test *P* value for the FV model was significant, indicating that the model is not well calibrated and not useful in predicting FV. The Hosmer-Lemeshow goodness-of-fit test *P* value for the HFV model was 0.14. Accuracy of the model as obtained by the AUC ROC was 0.79 (95% CI, 0.76–0.82).

## Discussion

Since no univocal definition of high ED utilization exists in the literature [2], we used the thresholds recently developed by Doupe et al. [6]. They suggested that patient characteristics changed meaningfully at a breakpoint of 7 ED visits per year, thereby providing an objective threshold [6].

We assessed 244 individual FV and HFV (244 of 51,272 = 0.5% of total ED patients in one year), who presented to the two EDs on 2,338 occasions (2,338 of 71,565 = 3.3% of total ED consultations in one year). FV and HFV together in this study are a low percentage (0.5%) compared to another study using the same thresholds, where FV composed 2.1% of users and 9.9% of ED visits, whereas HFV composed 0.2% and 3.6% of users and visits, respectively [6]. It is possible that the strong primary care network in the Netherlands prevents part of the ED visits. Since practically all Dutch citizens have a general practitioner (GP) and GP services are available 24/7, patients may present to their GP instead of at the ED.

FV were significantly older than patients with single visits (47.5 vs. 39.0 years), but HFV were not. Comparing our findings with other studies is difficult: we found a study indicating that the common FV is 35 years of age [10], as well as a study claiming that as individuals get older, the risk of high ED utilization increases slightly [12]. In our study, FV were equally likely to be male or female. Results in the existing literature are equivocal; in some studies, women were disproportionately associated with high ED utilization [1,2,24] but the contrary has also been observed [6,8,11]. Besides age and gender, literature also shows some variation in presenting complaints observed by study site. Coinciding with our findings, abdominal complaints [10], shortness of breath [4,9,21], and mental illness [1,5,7,21] tend to be the most common. Psychiatric morbidity has been found to be a significant predictor of high ED utilization [1,5,6,8,9,13,21]. In our study, the odds of being a FV or a HFV (versus a patient with one ED visit) was about 3- to 7-fold greater for patients attending with a psychiatric problem as the chief complaint.

In other studies, most frequent visits occurred during the evening or night shifts [3,10]. In our study, most of the FV and HFV arrived during the day or evening shift. However, when corrected for other variables, arriving during the night shift was indicative for high ED utilization. Frequent ED use during so called off-hours is sometimes thought to be indicative of inadequate primary care [10]; however, in the Netherlands most people are registered with a local GP and out-of-hours GP cooperatives are available 24/7.

Although heterogeneity is assumed amongst the subgroups of FV and HFV, in our study, HFV were not very different from FV: they presented with the same problems and both were less likely to be self-referred compared with single visit patients. We found differences mainly in admission rates and ambulance-use; HFV were more often brought in by ambulance but less often admitted for further analysis. However, the validity of our conclusions regarding the HFV, being a very small group of patients (n = 12), may be unreliable and differences between FV and HFV, and between HFV and patients with a single visit should be further examined using a larger sample of HFV.

Most studies indicate that patients with high ED utilization are usually sick patients with chronic illness associated with high admission rates [1,2,7]. To corroborate this, our FV and HFV were less often assigned to the non-urgent or standard triage level than patients with single visits, and they were less likely to be self-referrals and FV had higher admission rates compared to patients with single visits. These findings suggest that FV present with more alarming symptoms. We recommend building alerts into the ED information system, drawing attention of the treating physician when a patient presents to the ED for the seventh time within a year. If hospital admission is not indicated for that particular patient, at least an appointment at the outpatient clinic should be considered.

### Limitations

First, we were not able to account for some of the socio-economic factors that are known to influence high ED utilization, such as race, alcohol dependence, homelessness, and insurance coverage [3,5,10,17,21]. More work is needed to search for other potential risk factors not captured in this study.

Second, the finding that FV had higher admission rates than patients with single visits should be considered with great caution and warrants further investigation. When patients present for the third or fourth time within a few days, physicians tend to admit this patient for further analysis, so bias by frequency of presentation is possible.

Third, we had no data on our patients’ medical diagnoses and thus we were not able to compare our findings regarding psychiatric co-morbidity with other studies. Instead of diagnoses, we used chief complaints based on the triage flow charts. The use of chief complaints was sufficient for our purpose (identifying factors associated with high ED utilization) and certainly more feasible than using medical diagnoses.

Fourth, we had no data on patients’ use of other EDs in the surrounding area. FV and HFV are prone to use more than one health care service [18], so the rate of their ED use may in fact be an underestimation.

Finally, our findings may have limited generalizability because of the cultural, social, and health care delivery characteristics of our population.

## Conclusions

Based on this two-ED study, in the Netherlands high ED utilization seems to be less of a problem than outlined in international literature. No major differences were found between FV and HFV. Both presented with shortness of breath, abdominal pain, urinary tract problems, or psychiatric disorders more often compared to patients with single ED visits. FV were more likely to be admitted than patients with single visits. Our study supports the notion that most patients with high ED utilization visit the ED for significant medical problems.

## Abbreviations

AUC ROC: Area under the receiver operating curve; CI: Confidence interval; ED: Emergency department; FV: Frequent visitor; GP: General practitioner; HFV: Highly frequent visitor; LWBS: Leaving without being seen; OR: Odds ratio.

## Competing interests

The authors declare that there are no competing interests.

## Authors’ contributions

CvdL had full access to all of the data in the study and takes responsibility for the integrity of the data and the accuracy of the data analysis. Study concept & design: CvdL, CvdB, NvdL, AR, and CB. Acquisition of the data: CvdL. Analysis and interpretation of data: CvdL and CvdB. Drafting of the manuscript: CvdL, CvdB, NvdL, and AR. Critical revision of the manuscript for important intellectual content: CvdB and CB. All authors read and approved the final manuscript.

## References

[B1] Fuda KK Immekus R Frequent users of Massachusetts emergency departments: a statewide analysis Ann Emerg Med 2006 7 9 16 16781915 10.1016/j.annemergmed.2006.03.001

[B2] LaCalle E Rabin E Frequent users of emergency departments: the myths, the data, and the policy implications Ann Emerg Med 2010 7 42 48 10.1016/j.annemergmed.2010.01.032 20346540

[B3] Althaus F Stucki S Guyot S Trueb L Moschetti K Daeppen JB Bodenmann P Characteristics of highly frequent users of a Swiss academic emergency department: a retrospective consecutive case series Eur J Emerg Med 2013 7 6 413 419 10.1097/MEJ.0b013e32835e078e 23337095

[B4] Dent AW Phillips GA Chenhall AJ McGregor LR The heaviest repeat users of an inner city emergency department are not general practice patients Emerg Med (Fremantle) 2003 7 322 329 14631698 10.1046/j.1442-2026.2003.00470.x

[B5] Doran KM Raven MC Rosenheck RA What drives frequent emergency department use in an integrated health system? National data from the Veterans Health Administration Ann Emerg Med 2013 7 2 151 159 10.1016/j.annemergmed.2013.02.016 23582617

[B6] Doupe MB Palatnick W Day S Chateau D Soodeen RA Burchill C Derksen S Frequent users of emergency departments: developing standard definitions and defining prominent risk factors Ann Emerg Med 2012 7 24 32 10.1016/j.annemergmed.2011.11.036 22305330

[B7] Hunt KA Weber EJ Showstack JA Colby DC Callaham ML Characteristics of frequent users of emergency departments Ann Emerg Med 2006 7 1 8 16781914 10.1016/j.annemergmed.2005.12.030

[B8] Jelinek GA Jiwa M Gibson NP Lynch AM Frequent attenders at emergency departments: a linked-data population study of adult patients Med J Aust 2008 7 552 556 19012551 10.5694/j.1326-5377.2008.tb02177.x

[B9] Lacalle EJ Rabin EJ Genes NG High-frequency users of emergency department care J Emerg Med 2013 7 6 1167 1173 10.1016/j.jemermed.2012.11.042 23473816

[B10] Milbrett P Halm M Characteristics and predictors of frequent utilization of emergency services J Emerg Nurs 2009 7 191 198 10.1016/j.jen.2008.04.032 19446122

[B11] Moore L Deehan A Seed P Jones R Characteristics of frequent attenders in an emergency department: analysis of 1-year attendance data Emerg Med J 2009 7 263 267 10.1136/emj.2008.059428 19307386

[B12] Peppe EM Mays JW Chang HC Becker E DiJulio B Characteristics of Frequent Emergency Department Users 2007 Menlo Park, CA: The Henry J. Kaiser Family Foundation

[B13] Pillow MT Doctor S Brown S Carter K Mulliken R An emergency department-initiated, web-based, multidisciplinary approach to decreasing emergency department visits by the top frequent visitors using patient care plans J Emerg Med 2013 7 853 860 10.1016/j.jemermed.2012.08.020 23102594

[B14] Pines JM Asplin BR Kaji AH Lowe RA Magid DJ Raven M Weber EJ Yealy DM Frequent users of emergency department services: gaps in knowledge and a proposed research agenda Acad Emerg Med 2011 7 e64 e69 10.1111/j.1553-2712.2011.01086.x 21676051

[B15] Ruger JP Richter CJ Spitznagel EL Lewis LM Analysis of costs, length of stay, and utilization of emergency department services by frequent users: implications for health policy Acad Emerg Med 2004 7 1311 1317 10.1111/j.1553-2712.2004.tb01919.x 15576522

[B16] Sandoval E Smith S Walter J Schuman SA Olson MP Striefler R Brown S Hickner J A comparison of frequent and infrequent visitors to an urban emergency department J Emerg Med 2010 7 115 121 10.1016/j.jemermed.2007.09.042 18462906

[B17] Wajnberg A Hwang U Torres L Yang S Characteristics of frequent geriatric users of an urban emergency department J Emerg Med 2012 7 376 381 10.1016/j.jemermed.2011.06.056 22040771 PMC13458729

[B18] Han A Ospina M Blitz SB Strome T Rowe BH Patients presenting to the emergency department: the use of other health care services and reasons for presentation CJEM 2007 7 428 434 18072988 10.1017/s1481803500015451

[B19] Capp R Rosenthal MS Desai MM Kelley L Borgstrom C Cobbs-Lomax DL Simonette P Spatz S Characteristics of Medicaid enrollees with frequent ED use Am J Emerg Med 2013 7 9 1333 1337 10.1016/j.ajem.2013.05.050 23850143

[B20] Martin GB Stokes-Buzzelli SA Peltzer-Jones JM Schultz LR Ten years of frequent users in an urban emergency department West J Emerg Med 2013 7 243 246 10.5811/westjem.2012.5.11853 23687543 PMC3656705

[B21] Dent A Hunter G Webster AP The impact of frequent attenders on a UK emergency department Eur J Emerg Med 2010 7 332 336 10.1097/MEJ.0b013e328335623d 20038842

[B22] Mackway-Jones K Marsden J Windle J Emergency Triage 2005 London: BMJ Publishing Group

[B23] Hosmer D Lemeshow S Applied Logistic Regression 2000 Hoboken, NJ: John Wiley & Sons

[B24] LeDuc K Rosebrook H Rannie M Gao D Pediatric emergency department recidivism: demographic characteristics and diagnostic predictors J Emerg Nurs 2006 7 131 138 10.1016/j.jen.2005.11.005 16580475

